# Prevalence and correlates of current cigarette smoking among transgender women in Argentina

**DOI:** 10.3389/fpubh.2023.1279969

**Published:** 2023-12-05

**Authors:** Francisco Cartujano-Barrera, Raul M. Mejia, Pablo D. Radusky, Nadir Cardozo, Mariana Duarte, Solange Fabian, Romina Caballero, Virginia Zalazar, Alixida Ramos-Pibernus, Ash B. Alpert, Ana Paula Cupertino, Claudia Frola, Ines Aristegui

**Affiliations:** ^1^Department of Public Health Sciences, University of Rochester Medical Center, Rochester, NY, United States; ^2^Division of Implementation Research, Fundación Huésped, Buenos Aires, Argentina; ^3^Department of Ambulatory Care, Universidad de Buenos Aires, Buenos Aires, Argentina; ^4^Asociación de Travestis, Transexuales y Transgéneros de Argentina, Buenos Aires, Argentina; ^5^Casa Trans, Buenos Aires, Argentina; ^6^Hotel Gondolín, Buenos Aires, Argentina; ^7^School of Behavioral and Brain Sciences, Ponce Health Sciences University, Ponce, PR, United States; ^8^Division of Hematology, Department of Medicine, Yale School of Medicine, New Haven, CT, United States; ^9^Department of Surgery, University of Rochester Medical Center, Rochester, NY, United States

**Keywords:** smoking, tobacco, transgender individuals, transgender women, Argentina

## Abstract

**Objective:**

To assess the prevalence of current cigarette smoking among transgender women in Argentina, and to examine the unique associations of current cigarette smoking with demographic and psychosocial factors.

**Methods:**

This study is a secondary data analysis of the TransCITAR – a prospective cohort study of transgender individuals living in Buenos Aires, Argentina – baseline data. The baseline survey collected information on sociodemographic characteristics, perceived health status, depressive symptoms, suicide attempts, current cigarette smoking, alcohol use disorder, and substance use. Participants were also asked about lifetime experiences of physical and sexual violence perpetrated by partners, clients and/or the police, and experiences of gender identity stigma in the past year from healthcare workers and the police. Lastly, participants were asked if they had ever been arrested. Fisher’s exact test was used to compare proportions in categorical variables and student *t*-test was used for continuous variables. Significant associations with current cigarette smoking were tested in a logistic regression model adjusted for all significant associations.

**Results:**

A total of 41.7% of participants (*n* = 393) reported current cigarette smoking. Compared to their non-smoking counterparts, participants who reported current cigarette smoking (1) had completed less education, (2) were more likely to be born in Argentina, (3) more likely to had migrated to Buenos Aires from other parts of the country, (4) more likely to report a history of sex work, (5) more likely to perceive their health as excellent, (6) more likely to screen positive for hazardous alcohol drinking, (7) more likely to report any substance and cocaine use in the past year, (8) more likely to experience gender identity stigma from the police in the past year, and (9) more likely to being arrested in their lifetime (all *p*’s < 0.05). After controlling for all significant associations, education level of less than high school (AOR = 1.79, 95% CI 1.02–2.12), hazardous drinking (AOR = 2.65, 95% CI 1.30–5.37), and any substance use in the last year (AOR = 2.14, 95% CI 1.16–3.94) were positively and independently associated with current cigarette smoking.

**Conclusion:**

Among transgender women in Argentina, current cigarette smoking was more than double the rate for cisgender women. Current cigarette smoking was associated with education, hazardous drinking, and any drug use. These results will inform future smoking cessation interventions among transgender women in Argentina.

## 1 Introduction

Low- and middle-income countries (LMICs), including Argentina – an upper middle-income country – have not benefited from the decline in smoking-related deaths that high-income countries have experience since the 1980s (1, 2). In 2003, Argentina signed the Framework Convention for Tobacco Control (FCTC) (3), an international treaty that aims to reduce the demand and supply of tobacco (4). While this treaty has still not yet been ratified by the National Congress, other tobacco control policies implemented in Argentina (e.g., smoke-free environments, excise tax on cigarettes) have resulted in a decline of smoking prevalence by 50% between 2004 and 2012 (from 37 to 22.1%) (5–7). However, since 2012, the smoking prevalence in Argentina has remained unchanged (7, 8). Today, Argentina has one the highest smoking rates in Latin America: 6 million adults (22%) currently smoke (18% of women and 26% of men) (7). Consequently, smoking is directly responsible for nearly 1 million years of healthy life lost each year in Argentina and is the cause of 13.2% of all deaths in people over the age of 35, a total of 44,851 preventable deaths annually (8). To decrease the current smoking rates in LMICs (e.g., Argentina) and their public health impact, it is imperative to develop and implement interventions that reach and engage individuals in smoking cessation treatment.

*Transgender* is an umbrella term for persons whose gender identity, gender expression, and/or behavior differs from that typically associated with the sex to which they were assigned at birth (9). Research on the prevalence of cigarette smoking among transgender individuals living in LMICs is limited, but existing evidence suggests that rates are higher than the general population (10, 11). For example, a community-based cross-sectional study with 734 transgender adults living in India reported that 64.4% of study participants currently used a form of tobacco (10). Another example is a community-based cross-sectional study with 206 transgender and non-binary youth (ages 16–25) living in Brazil that reported a 14.1% prevalence of cigarette smoking (11). The minority stress model (MSM) may explain the higher prevalence of cigarette smoking among transgender individuals. MSM proposes that, along with experiencing the everyday stressors shared by the general population, there are certain stressors unique to minority groups (e.g., external and internal gender minority stress) (12). Additional research is needed to assess the prevalence and correlates of cigarette smoking among transgender individuals living in different LMICs – especially research that considers the diverse cultural, legal (e.g., transgender rights), clinical (e.g., healthcare access), and economic settings of different LMICs (13). The purpose of the present study was thus two-fold: (1) to assess the prevalence of current cigarette smoking among transgender women in Argentina, and (2) to examine the unique associations of current cigarette smoking with demographic and psychosocial factors. These results will inform future smoking cessation interventions among transgender women in Argentina.

## 2 Methods

### 2.1 Study design

This study is a secondary data analysis of the *TransCITAR* baseline data. *TransCITAR* is a prospective cohort study of transgender individuals living in the metropolitan area of Buenos Aires, Argentina. The details of *TransCITAR* have been described elsewhere (14). Study procedures were approved and monitored by Fundacion Huesped’s Institutional Review Board (IRB00002014 # FH-41).

### 2.2 Participants

Transgender individuals were recruited by transgender peer navigators using community-based recruitment strategies (e.g., conducting study presentations, distributing flyers, etc.) in venues that serve transgender individuals (e.g., recreation spaces for transgender individuals, hotels that serve as home for transgender individuals, organizations providing testing and treatment of sexually transmitted diseases), in a research clinic that provides gender-affirming care, and word of mouth from participants. Recruitment started in September 2019 and ended in September 2022. Individuals were eligible if they self-reported a gender identity different from that commonly associated with their sex assigned at birth and were older than 18 years old. Written informed consent was obtained from participants prior to study procedures. Participants did not receive compensation for their participation in the study.

### 2.3 Measures

The *TransCITAR* baseline survey was developed with members of the transgender community and collected information on sociodemographic characteristics [e.g., gender identity, age, educational level, place of birth, social and income assistance, housing situation, and engagement in sex work]. The baseline survey additionally assessed participants’ perceived health status (i.e., poor, fair, good, very good, or excellent) (15), depressive symptoms (measured by the Center for Epidemiologic Studies Depression Scale) (16), and suicide attempts. Participants were asked if they currently smoke or have smoked cigarettes (Do you/did you smoke cigarettes? Yes, currently; yes, in the past; never). Alcohol use disorder and substance use were assessed with the Alcohol Use Disorder Identification Test (AUDIT) and the Drug Abuse Screening Test (DAST-10), respectively (17–19). Assessment of substance use included use of marijuana, cocaine, cocaine paste (regionally known as *paco*, a crude extract of the coca leaf which contains 40 to 91% cocaine freebase along with companion coca alkaloids and varying quantities of benzoic acid, methanol, and kerosene), ecstasy, heroin, lysergic acid diethylamide (LSD), crack, popper, and amphetamines. Participants were also asked about lifetime experiences of physical and sexual violence perpetrated by partners, clients and/or the police. They were also asked about lifetime experiences of gender identity stigma perpetrated by healthcare workers and the police. Lastly, participants were asked if they had ever been arrested. Study surveys were administered by transgender peer navigators.

### 2.4 Analyses

Only participants who self-identified as transgender women were included in the analysis. This determination was due to their high representation in the *TransCITAR* study (84% of the *TransCITAR* participants). Moreover, previous studies conducted in Argentina have identified that transgender men and non-binary individuals have different demographic and psychosocial factors compared to transgender women (20). For example, compared to transgender women, transgender men have higher educational levels and lower levels of unstable housing (20).

Frequencies and percentages were calculated for categorical variables, and means and standard deviations for continuous variables. Fisher’s exact test was used to compare proportions in categorical variables and crude odds ratios (OR) were reported, and student t-test was used for continuous variables. Significant associations with current cigarette smoking (at the *p* < 0.05 level) were tested in an unadjusted logistic regression model, followed by a logistic regression model adjusted for all significant associations. Statistical analyses were performed using the Statistical Package for the Social Sciences v29.0 (SPSS; IBM, 2022).

### 2.5 Sample size

Estimating a current cigarette smoking rate of 40% in a sample of 356 participants insures a margin of error of 5%. Moreover, with the general guideline of 10 events per variable, 356 participants is an adequate sample size for a logistic regression model accommodating 10 variables.

## 3 Results

### 3.1 Sample

A total of 393 transgender women were included in the study. Participants’ mean age was 32.0 years old (SD 8.96). Half of the participants (50.6%) had not completed high school, 30.8% were born in a country other than Argentina, and 68.9% migrated to Buenos Aires from other parts of the country. Almost half of participants reported receiving social and income assistance (46.7%) and unstable housing (42.0%). More than three-quarter of participants (78.4%) reported participating in sex work at some point in their lives. Most participants (76.3%) perceived their health as good, very good, or excellent. A third of participants (32.8%) met criteria for depression, and one quarter (25.4%) reported a past suicide attempt. A total of 19.9% of participants screened positive for hazardous alcohol drinking. In the prior year, 63.1% of participants reported using at least one substance, 46.1% reported marijuana use, 29.8% reported cocaine use, and 5.1% reported ecstasy use. Half of participants (52.7%) reported physical violence perpetrated by partners, clients and/or the police, and 29.6% reported sexual violence from those same groups. Moreover, 23.7 and 29.8% of participants reported experiencing gender identity stigma from the police and healthcare workers, respectively. Less than half (41.2%) of participants reported a prior arrest (Table 1).

**Table 1:** Characteristics of transgender women and current cigarette smoking (*n*= 393).

Characteristics	Total n (%)	No current cigarette smoking n (%)	Current cigarette smoking n (%)	*p*
	393	229 (58.3)	164 (41.7)	
Age, M (SD)	32.0 (8.96)	31.9 (8.94)	32.1 (9.04)	0.827
Educational level				**0.001**
Less than high school	199 (50.6)	99 (43.2)	100 (61.0)	
High school completed or more	194 (49.4)	130 (56.8)	64 (39.0)	
Born in different country, yes	121 (30.8)	83 (36.2)	38 (23.2)	**0.006**
Internal migration, yes	190 (69.9)	94 (64.4)	96 (76.2)	**0.034**
Receive social and income assistance, yes	183 (46.7)	97 (42.5)	86 (52.4)	0.053
Unstable housing, yes	165 (42.0)	97 (42.4)	68 (41.5)	0.859
Lifetime sex work, yes	308 (78.4)	169 (73.8)	139 (84.8)	**0.009**
Perceived health status				**0.008**
Poor	14 (3.6)	9 (3.9)	5 (3.0)	
Fair	79 (20.1)	47 (20.5)	32 (19.5)	
Good	140 (35.6)	88 (38.4)	52 (31.7)	
Very good	100 (25.4)	63 (27.5)	37 (22.6)	
Excellent	60 (15.3)	22 (9.6)	38 (23.2)	
HIV status, positive	170 (43.3)	92 (40.2)	78 (47.6)	0.054
Depression, positive^a^	129 (32.8)	75 (32.8)	54 (32.9)	0.971
Past suicide attempts, yes	100 (25.4)	59 (25.8)	40 (24.4)	0.757
Hazardous alcohol drinking, yes^b^	78 (19.9)	30 (13.2)	48 (29.3)	**<0.001**
Any substance use, yes, past year	248 (63.1)	123 (53.7)	125 (76.2)	**<0.001**
Marijuana use, yes, past year	181 (46.1)	73 (40.8)	108 (50.5)	0.055
Cocaine use, yes, past year	117 (29.8)	29 (16.2)	88 (41.1)	**<0.001**
Ectasis use, yes, past year	20 (5.1)	5 (2.8)	15 (7.0)	0.058
Physical violence, lifetime, yes	155 (52.7)	82 (50.0)	63 (56.2)	0.294
Sexual violence, lifetime, yes	85 (29.6)	45 (28.5)	40 (31.0)	0.641
GIS from the police, past year, yes	93 (23.7)	42 (18.3)	51 (31.1)	**0.003**
GIS from healthcare workers, past year, yes	117 (29.8)	117 (29.7)	117 (29.7)	0.626
Arrested by the police, lifetime, yes	162 (41.2)	83 (36.2)	79 (48.2)	**0.018**

Bold indicates statistical significance at *p* < 0.05.

^a^CES-D score ≥ 16.

^b^AUDIT score ≥ 8. GIS: gender identity stigma.

### 3.2 Prevalence and correlates of current cigarette smoking

A total of 41.7% of participants reported current cigarette smoking. The median age of cigarette smoking initiation was 16 years (IQR: 15–18). Compared to their non-smoking counterparts, participants who reported current cigarette smoking (1) had completed less education, (2) were more likely to be born in Argentina, (3) more likely to have migrated to Buenos Aires from other parts of the country, (4) more likely to report a history of sex work, (5) more likely to perceive their health as excellent, (6) more likely to screen positive for hazardous alcohol drinking, (7) more likely to report any substance and cocaine use in the past year, (8) more likely to experience gender identity stigma from the police in the past year, and (9) more likely to being arrested in their lifetimes (Table 1; all *p*’s < 0.05).

Results of the unadjusted logistic regression model are presented in Table 2. After controlling for all significant associations, education level of less than high school (AOR = 1.79, 95% CI 1.02–2.12), hazardous drinking (AOR = 2.65, 95% CI 1.30–5.37), and any substance use in the last year (AOR = 2.14, 95% CI 1.16–3.94) were positively and independently associated with current cigarette smoking.

**Table 2:** Logistic regression models of current cigarette smoking (*n* = 393).

	Model 1 Unadjusted (OR, 95% CI)	Model 2 Adjusted (AOR, 95% CI)
Education level: Less than high school	**1.347 (1,135–1.598)**	**1.788 (1.023–2.124)**
Perceived health status: bad/regular	**1.111 (0.692–1.785)**	0.848 (0.451–1.597)
Born in different country, yes	**1.475 (1.101–1.977)**	---
Internal migration, yes	**1.282 (1.030–1.595)**	1.128 (0.592–2.146)
Lifetime sex work, yes	**1.286 (1.085–1.526)**	1.076 (0.484–2.391)
Hazardous alcohol drinking, yes^a^	**1.639 (1.223–2.198)**	**2.646 (1.303–5.373)**
Any substance use, past year, yes	**2.767 (1.781–4.300)**	**2.138 (1.164–3.935)**
Cocaine use, past year, yes	**1.733 (1.358–2.212)**	1.355 (0.790–2.323)
GIS from the police, past year, yes	**1.380 (1.085–1.756)**	1.431 (0.641–3.193)
Arrested by the police, lifetime, yes	**1.234 (1.031–1.476)**	1.047 (0.503–2.183)

Bold indicates statistical significance at *p* < 0.05.

## 4 Discussion

To the best of our knowledge, this is the first study to assess the prevalence of current cigarette smoking among transgender women in Argentina, and to examine the unique associations of current cigarette smoking with demographic and psychosocial factors. In this study, 41.7% of transgender women reported current cigarette smoking, more than double the rate for cisgender women (i.e., individuals who were assigned female at birth and identify as a woman) (7). Univariate analyses demonstrated that current cigarette smoking was significantly associated with education, external and internal migration, sex work, alcohol and drug use, experiencing gender identity stigma from the police, and being arrested. However, in the final multivariable model, only education, hazardous drinking, and any drug use remained associated with higher odds of current cigarette smoking.

In 2021, Wolford-Clevenger et al. conducted a systematic review of correlates of tobacco and nicotine use among transgender and gender diverse people guided by the MSM (21). Findings from our study support prior research demonstrating that police discrimination (22), indicators of poverty (e.g., less completed education levels) (23–25), and indicators of stress (e.g., alcohol and drug use) (23–25), are associated with an increased likelihood of cigarette use. However, our findings challenge prior research by indicating that engagement in sex work was not associated with cigarette use (23). Importantly, our findings shed light on new identity-related factors of the MSM (e.g., external and internal migration) that are correlated to current cigarette smoking. Specifically, our study findings show that external migration (i.e., living in a country different from the country of birth) was associated with decreased likelihood of cigarette use. Among transgender individuals, external migration due to gender identity-based stigma and oppression experienced in their home country has been reported (26, 27). One possible explanation that external migration is associated with a decreased likelihood of cigarette use is that, in this study, the majority of participants who emigrated to Argentina were born in countries with lower prevalence of cigarette use compared to Argentina (e.g., Paraguay) (28). Moreover, external migration has been reported as a protective factor against smoking in other countries (e.g., the United States of America) in the general population (29). Interestingly, internal migration (i.e., living in a city different from the city of birth) was associated with increased likelihood of cigarette use. One possible explanation for this phenomenon is that, in this study, the majority of participants who migrated to Buenos Aires came from the northwest region of Argentina (e.g., Jujuy) – a region that produces tobacco (30). More research is needed to understand the mechanisms underpinning these correlates.

While assessing the correlates of current cigarette smoking among transgender women in Argentina, it is important to note that Argentina has been praised for having one of the world’s most progressive transgender rights laws (31, 32). In 2012, Argentina passed the *Ley de Identidad de Género* (Spanish for “Gender Identity Law”), which allows transgender individuals to be treated according to their gender identity and have their personal documents registered with the appropriate name and gender (31). Under this comprehensive law, modifying personal data is free and does not require a lawyer (31). Moreover, the *Ley de Identidad de Género* mandates gender affirming treatments and procedures to be included in the *Programa Médico Obligatorio* (Spanish for “Compulsory Medical Program”), which guarantees medical coverage throughout the public and private health systems (31). Despite the existence of this law, 29% of participants – regardless of smoking status – reported experiencing gender identity stigma from healthcare workers in the past. Recently, transgender activists passed the *Cupo Laboral Travesti Trans* (Spanish for “Transgender Job Quota Law”) – a law reserving 1% of Argentina’s public sector jobs for transgender individuals (33). The *Cupo Laboral Travesti Trans* aims to reduce the high levels of social vulnerability (e.g., engagement in sex work and housing instability) faced by transgender individuals in Argentina (33). These laws, if correctly implemented, have the potential to influence cigarette smoking indirectly by addressing the social determinants of health among transgender individuals. The *TransCITAR* study has the potential to evaluate the longitudinal impact of these comprehensive laws on the social determinants of health and, consequently, the health and well-being of transgender individuals.

### 4.1 Strengths and limitations

The study has several methodological limitations. First, this study purposely focused on transgender women given their high representation in the *TransCITAR* study (84% of the *TransCITAR* participants). Future studies with appropriate representation of transgender men and non-binary individuals should assess the prevalence and correlates of current cigarette smoking among these communities. Second, as there are no official registries of transgender women in Argentina, the recruitment strategy was convenience sampling and thus findings may not be representative of transgender women in Argentina. Third, the study was solely conducted in Buenos Aires, limiting participation by transgender individuals from other parts of Argentina. Fourth, the study did not assess the number of cigarettes smoked per day nor type of cigarettes (e.g., menthol or non-menthol cigarettes), limiting the ability to examine whether different levels of cigarette smoking were related to different demographic and psychosocial factors. Fifth, data are self-reported and there is a possibility that participants felt compelled to offer socially desirable responses. Lastly, as a cross-sectional investigation of the *TransCITAR* baseline data, it was not possible to determine the causal nature of any of the observed associations.

Despite the methodological limitations, this study has several strengths. First, this study is among a small but growing number of reports on the prevalence and correlates of current cigarette smoking among transgender individuals in LMICs. This information is urgently needed to develop and implement interventions to decrease the current smoking rates in LMICs. Second, this study builds upon an established history of research with the transgender community (34–39). Third, this work is grounded in principles of community-based participatory research (CPBR). CBPR is a partnership approach to research that involves community members, organizational representatives, and researchers across all phases of research (40). Study results were reviewed and discussed with members of the transgender community. Lastly, this study benefited from an adequate sample size to assess the prevalence of current cigarette smoking among transgender women in Argentina, and to examine the unique associations of current cigarette smoking with demographic and psychosocial factors.

## 5 Conclusion

Among transgender women in Argentina, current cigarette smoking was more than double the rate for cisgender women. Current cigarette smoking was associated with education, hazardous drinking, and any drug use. These results will inform future smoking cessation interventions among transgender women in Argentina.

## Data availability statement

The original contributions presented in the study are included in the article/supplementary material, further inquiries can be directed to the corresponding author/s.

## Ethics statement

The studies involving humans were approved by the Fundacion Huesped’s Institutional Review Board (IRB00002014 # FH-41). The studies were conducted in accordance with the local legislation and institutional requirements. The participants provided their written informed consent to participate in this study.

## Author contributions

FC-B: Conceptualization, Writing – original draft. RM: Conceptualization, Writing – original draft. PR: Project administration, Validation, Writing – review & editing. NC: Project administration, Writing – review & editing. MD: Project administration, Writing – review & editing. SF: Project administration, Writing – review & editing. RC: Formal analysis, Writing – review & editing. VZ: Project administration, Writing – review & editing. AR-P: Writing – review & editing. AA: Writing – review & editing. AC: Writing – review & editing. CF: Writing – review & editing. IA: Conceptualization, Formal analysis, Writing – original draft.

## References

[1] Drope J Schluger NW Cahn Z Drope J Hamill S Islami F. (2018). American Cancer Society and vital strategies. Available at: https://pngcancerfoundation.org/wp-content/uploads/2021/06/TobaccoAtlas_6thEdition_LoRes.pdf (Accessed August 18, 2023).

[2] The World Bank. Data for upper middle income, Argentina. (2022). The World Bank. Available at: https://dataworldbankorg/?locations=XT-AR (Accessed August 18, 2023).

[3] Alcaraz A Caporale J Bardach A Augustovski F Pichon-Riviere A. Carga de enfermedad atribuible al uso de tabaco en Argentina y potencial impacto del aumento de precio a través de impuestos. Rev Panam Salud Publica. (2016) 40:204–12. PMID: 28001195

[4] World Health Organization. (2003). WHO framework convention on tobacco control. World Health Organization. Available at: https://fctc.who.int/who-fctc/overview (Accessed August 18, 2023).

[5] Konfino J Ferrante D Mejia R Coxson P Moran A Goldman L. Impact on cardiovascular disease events of the implementation of Argentina's national tobacco control law. Tob Control. (2014) 23:e6. doi: 10.1136/tobaccocontrol-2012-050599, PMID: 23092886 PMC4026283

[6] Ministerio de Salud de la Nacion. (2012). GATS/Encuesta Mundial de Tabaquismo en Adultos. Available at: https://bancos.salud.gob.ar/sites/default/files/2020-02/gats_encuesta-mundial-tabaquismo-adultos_2012_resumen.pdf (Accessed August 18, 2023).

[7] Ministerio de Salud y Desarrollo Social. (2018). 4ta Encuesta Nacional de Factores de Riesgo. https://bancos.salud.gob.ar/sites/default/files/2020-01/4ta-encuesta-nacional-factores-riesgo_2019_principales-resultados.pdf (Accessed August 18, 2023).

[8] Pichon-Riviere A Alcaraz A Palacios A Rodríguez B Reynales-Shigematsu LM Pinto M. The health and economic burden of smoking in 12 Latin American countries and the potential effect of increasing tobacco taxes: an economic modelling study. Lancet Glob Health. (2020) 8:e1282–94. doi: 10.1016/S2214-109X(20)30311-9, PMID: 32971051

[9] What does transgender mean? (2023). American Psychological Association. Available at: https://www.apa.org/topics/lgbtq/transgender (Accessed August 18, 2023).

[10] Sucharitha ST Pradeep R Vikram A Sivagurunathan C Ezhilvanan M Premkumar K. The pattern of tobacco use and the associated socio-demographic factors among transgenders living in Chennai City of Tamil Nadu, India. J Family Med Prim Care. (2022) 11:4452–9. doi: 10.4103/jfmpc.jfmpc_26_22, PMID: 36353038 PMC9638617

[11] Fontanari AMV Churchill S Schneider MA Soll B Costa AB Lobato MIR. Tobacco use among transgender and gender non-binary youth in Brazil. Cien Saude Colet. (2021) 26:5281–92. doi: 10.1590/1413-812320212611.3.35272019, PMID: 34787219

[12] Hendricks ML Testa RJ. A conceptual framework for clinical work with transgender and gender nonconforming clients: an adaptation of the minority stress model. Prof Psychol. (2012) 43:460–7. doi: 10.1037/a0029597

[13] Berg CJ Fong GT Thrasher JF Cohen JE Maziak W Lando H. The impact and relevance of tobacco control research in low-and middle-income countries globally and to the US. Addict Behav. (2018) 87:162–8. doi: 10.1016/j.addbeh.2018.07.012, PMID: 30041132 PMC6381392

[14] Frola C Caballero R Cesar C Frontini E Figueroa MI Perez CF. Transgender women baseline profile in TransCITAR: Trans-specific cohort in Argentina. Conference on retroviruses and opportunistic infections. Available at: https://www.croiconference.org/abstract/transgender-women-baseline-profile-in-transcitar-trans-specific-cohort-in-argentina/ (Accessed August 18, 2023).

[15] Vilagut G Ferrer M Rajmil L Rebollo P Permanyer-Miralda G Quintana JM. El Cuestionario de Salud SF-36 español: una década de experiencia y nuevos desarrollos [the Spanish version of the short form 36 health survey: a decade of experience and new developments]. Gac Sanit. (2005) 19:135–50. doi: 10.1157/13074369, PMID: 15860162

[16] Radloff LS. The CES-D scale: a self-report depression scale for research in the general population. Appl Psychol Meas. (1977) 1:385–401. doi: 10.1177/014662167700100306

[17] Saunders JB Aasland OG Babor TF de la Fuente JR Grant M. Development of the alcohol use disorders identification test (AUDIT): WHO collaborative project on early detection of persons with harmful alcohol consumption--II. Addiction. (1993) 88:791–804. doi: 10.1111/j.1360-0443.1993.tb02093.x, PMID: 8329970

[18] Skinner HA. The drug abuse screening test. Addict Behav. (1982) 7:363–71. doi: 10.1016/0306-4603(82)90005-3 7183189

[19] YudkoE LO Fouts A. A comprehensive review of the psychometric properties of the drug abuse screening test. J Subst Abus Treat. (2007) 32:189–98. doi: 10.1016/j.jsat.2006.08.002, PMID: 17306727

[20] de Travestis Asociaión, Transexuales y Transgéneros de Argentina, Fundación Huésped. Estudio sobre estado de la salud integral y derechos de masculinidades trans e identidades no binaries. Available at: https://huesped.org.ar/noticias/nuevo-estudio-sobre-masculinidades-trans-e-identidades-no-binaries/ (Accessed November 9, 2023).

[21] Wolford-Clevenger C Hill SV Cropsey K. Correlates of tobacco and nicotine use among transgender and gender diverse people: a systematic review guided by the minority stress model. Nicotine Tob Res. (2022) 24:444–52. doi: 10.1093/ntr/ntab159, PMID: 34375426 PMC11494233

[22] Lipperman-Kreda S Wilson I Hunt GP Annechino R Antin TMJ. Substance use among sexual and gender minorities: association with police discrimination and police mistrust. Sex Gend Policy. (2020) 3:92–104. doi: 10.1002/sgp2.12019, PMID: 34651132 PMC8513710

[23] Gamarel KE Mereish EH Manning D Iwamoto M Operario D Nemoto T. Minority stress, smoking patterns, and cessation attempts: findings from a community-sample of transgender women in the San Francisco Bay Area. Nicotine Tob Res. (2016) 18:306–13. doi: 10.1093/ntr/ntv066, PMID: 25782458 PMC4757930

[24] Shires DA Jaffee KD. Structural discrimination is associated with smoking status among a national sample of transgender individuals. Nicotine Tob Res. (2016) 18:1502–8. doi: 10.1093/ntr/ntv221, PMID: 26438646

[25] Kcomt L Evans-Polce RJ Veliz PT Boyd CJ McCabe SE. Use of cigarettes and e-cigarettes/vaping among transgender people: results from the 2015 U.S. transgender survey. Am J Prev Med. (2020) 59:538–47. doi: 10.1016/j.amepre.2020.03.027, PMID: 32826126 PMC7508765

[26] Cerezo A Morales A Quintero D Rothman S. Trans migrations: exploring life at the intersection of transgender identity and immigration. Psychol Sex Orientat Gend Divers. (2014) 1:170–80. doi: 10.1037/sgd0000031

[27] Toro-Alfonso J Ortiz ML Lugo KN. Sexualidades migrantes: La emigracion de hombres dominicanos gay. Caribb Stud. (2012) 40:59–80. doi: 10.1353/crb.2012.0017

[28] Global State of Tobacco Harm Reduction. (2023). Smoking, vaping, HTP, NRT, and snus in Paraguay. Available at: https://gsthr.org/countries/profile/pry/ (Accessed November 9, 2023).

[29] Acevedo-Garcia D Pan J Jun HJ Osypuk TL Emmons KM. The effect of immigrant generation on smoking. Soc Sci Med. (2005) 61:1223–42. doi: 10.1016/j.socscimed.2005.01.027 15970233

[30] de Agroindustria Secretaría. (2018). Producción de tabaco por provincia y departamento. Available at: https://www.magyp.gob.ar/sitio/areas/tabaco/produccion_mercados/interno/_archivos/000000_Producción%20Primaria/100001%20Produccion%20por%20Departamento%20Campaña%202017%20-%202018.pdf. Accessed November 9, 2023.

[31] de Cultura Ministerio. (2022). Ley de Identidad de Género: 10 años. Available at: https://www.argentina.gob.ar/noticias/ley-de-identidad-de-genero-10-anos. Accessed August 18, 2023.

[32] Socías ME Marshall BD Arístegui I Zalazar V Romero M Sued O. Towards full citizenship: correlates of engagement with the gender identity law among transwomen in Argentina. PLoS One. (2014) 9:e105402. doi: 10.1371/journal.pone.0105402, PMID: 25133547 PMC4136870

[33] Valente M. (2023). Transgender job quota law seen 'changing lives' in Argentina. Reuters. Available at: https://www.reuters.com/article/us-argentina-lgbt-lawmaking-trfn-idUSKCN2E11QV (Accessed August 18, 2023).

[34] Frola CE Aristegui I Figueroa MI Radusky PD Cardozo N Zalazar V. Retention among transgender women treated with dolutegravir associated with tenofovir/lamivudine or emtricitabine in Argentina: TransViiV study. PLoS One. (2023) 18:e0279996. doi: 10.1371/journal.pone.0279996, PMID: 36662723 PMC9858466

[35] Radusky PD Aristegui I Mandell LN Dell'Isola E Zalazar V Cardozo N. Examining factors associated with gender identity among individuals disengaged from HIV care in Argentina. Int J Behav Med. (2022) 29:69–77. doi: 10.1007/s12529-021-09998-6, PMID: 33954892 PMC8901250

[36] Zalazar V Frola CE Gun A Radusky PD Panis NK Cardozo NF. Acceptability of dual HIV/syphilis rapid test in community- and home-based testing strategy among transgender women in Buenos Aires, Argentina. Int J STD AIDS. (2021) 32:501–9. doi: 10.1177/0956462420979852, PMID: 33533303

[37] Zalazar V Aristegui I Socías ME Cardozo N Sued O Shannon K. Ethics and the treatment as prevention strategy among transgender women living with HIV in Argentina. Cult Health Sex. (2020) 23:674–89. doi: 10.1080/13691058.2020.1720821, PMID: 32213129

[38] Aristegui I Radusky PD Zalazar V Lucas M Sued O. Resources to cope with stigma related to HIV status, gender identity and sexual orientation in gay men and transgender women. J Health Psychol. (2018) 23:320–31. doi: 10.1177/1359105317736782, PMID: 29069922

[39] Aristegui I Radusky PD Zalazar V Romero M Schwartz J Sued O. Impact of the gender identity law in Argentinean transgender women. Int J Transgend. (2017) 18:446–56. doi: 10.1080/15532739.2017.1314796

[40] Israel BA Schulz AJ Parker EA Becker AB. Review of community-based research: assessing partnership approaches to improve public health. Annu Rev Public Health. (1998) 19:173–202. doi: 10.1146/annurev.publhealth.19.1.173 9611617

